# Ishophloroglucin A Isolated from *Ishige okamurae* Suppresses Melanogenesis Induced by α-MSH: In Vitro and In Vivo

**DOI:** 10.3390/md18090470

**Published:** 2020-09-17

**Authors:** Xining Li, Hye-Won Yang, Yunfei Jiang, Jae-Young Oh, You-Jin Jeon, Bomi Ryu

**Affiliations:** 1 Department of Marine Life Sciences, Jeju National University, Jeju 63243, Korea; lixn887@nenu.edu.cn (X.L.); koty221@naver.com (H.-W.Y.); jiangyunfei0310@gmail.com (Y.J.); ojy0724@naver.com (J.-Y.O.); 2 Marine Science Institute, Jeju National University, Jeju 63333, Korea

**Keywords:** Ishige okamurae, melanogenesis, α-MSH, zebrafish

## Abstract

Diphlorethohydroxycarmalol (DPHC) isolated from *Ishige okamurae* (IO) showed potential whitening effects against UV-B radiation. However, the components of IO as well as their molecular mechanism against α-melanocyte-stimulating hormone (α-MSH) have not yet been investigated. Thus, this study aimed to investigate the inhibitory effects of Ishophloroglucin A (IPA), a phlorotannin isolated from brown algae IO, and its crude extract (IOE), in melanogenesis in vivo in an α-MSH-induced zebrafish model and in B16F10 melanoma cells in vitro. Molecular docking studies of the phlorotannins were carried out to determine their inhibitory effects and to elucidate their mode of interaction with tyrosinase, a glycoprotein related to melanogenesis. In addition, morphological changes and melanin content decreased in the α-MSH-induced zebrafish model after IPA and IOE treatment. Furthermore, Western blotting results revealed that IPA upregulated the extracellular related protein expression in α-MSH-stimulated B16F10 cells. Hence, these results suggest that IPA isolated from IOE has a potential for use in the pharmaceutical and cosmetic industries.

## 1. Introduction

Marine algae have gained immense attention from the functional food, cosmetics, and pharmaceutical industries as they utilize various bioactive compounds such as phlorotannins, fucoidans, polysaccharides, and proteins [1,2,3,4,5,6,7]. According to a previous study, *Ishige okamurae* (IO) was screened for its inhibitory effects on tyrosinase activity [8]. Although its phlorotannin, diphlorethohydroxycarmalol (DPHC), was assessed for anti-melanogenesis activity induced by UV-B radiation in an in vitro model [8], no further studies were performed to explain the interaction between DPHC and melanogenesis-related proteins. In this study, a novel polyphenol compound, Ishophloroglucin A (IPA) [9], was evaluated for its interaction with tyrosinase in a molecular docking study, compared with that of DPHC.

Molecular docking is an efficient computational method to predict the potential binding mode of small molecules or ligands within the active site of a protein or receptor of a known three-dimensional structure for studying their interaction patterns or for drug design [10,11,12]. Previous studies have reported the interaction site and energy value of tyrosinase enzyme as investigated by molecular docking studies [13,14]. Functionally, tyrosinase is a rate-limiting enzyme that catalyzes a relatively slow rate of reaction in the signaling pathway and plays a pivotal role in melanin biosynthesis in specialized organelles called melanosomes, specifically synthesized by melanocytic cells [15]. Thus, the enzymatic activity of tyrosinase as modeled through in silico molecular docking simulations has been the target for the investigation of inhibitors to prevent cutaneous hyperpigmentary disorders. Based on this, in experimental and computational fields, many phenolic compounds or inhibitors possessing metal chelating ability have been widely studied as tyrosinase inhibitors [16].

Melanin primarily modulates skin color and acts as a protective pigment when the skin is exposed to UV radiation [17]. Exposure to UV radiation causes the release of α-melanocyte-stimulating hormone (α-MSH) from cutaneous keratinocytes and melanocytes to induce melanin synthesis and subsequently protect the skin from the damaging effects of solar radiation [18]. However, long-term exposure to UV radiation induces significant melanin synthesis by α-MSH, resulting in skin damage such as freckles, sunspots, and black spots on the skin epidermis, leading to DNA photodamage and skin cancer [19]. Following this, the administration of α-MSH into melanocytes has been widely studied to induce considerable changes in the tyrosinase activity for melanogenesis in melanoma [20].

Zebrafish display variations in skin color like humans, and are an animal model used for understanding the genetic origin of human skin color, connecting basic model organism biology with human genetics [21]. In addition, the pigments on the ventral and lateral regions of the zebrafish gastrula and their transparency during larval stages allow simple observation of the pigmentation process, providing an opportunity to develop viable models for understanding skin cell disorders resulting from defects in melanocyte development [22,23,24].

Melanin synthesis occurs via the activity of several melanogenesis-related proteins, such as tyrosinase, microphthalmia-associated transcription factor (MITF), tyrosinase-related protein-1 (Trp-2), and tyrosinase-related protein-1 (Trp-1) [15]. In this study, we aimed to investigate the inhibitory effect of IPA and IO crude extract (IOE) in tyrosinase activity and melanogenesis on α-MSH-induced zebrafish in vivo and B16F10 melanoma cells in vitro in order to evaluate their potential use in treating skin pigmentation and melanoma.

## 2. Results

### 2.1. Molecular Docking Study

**Figure 1:**
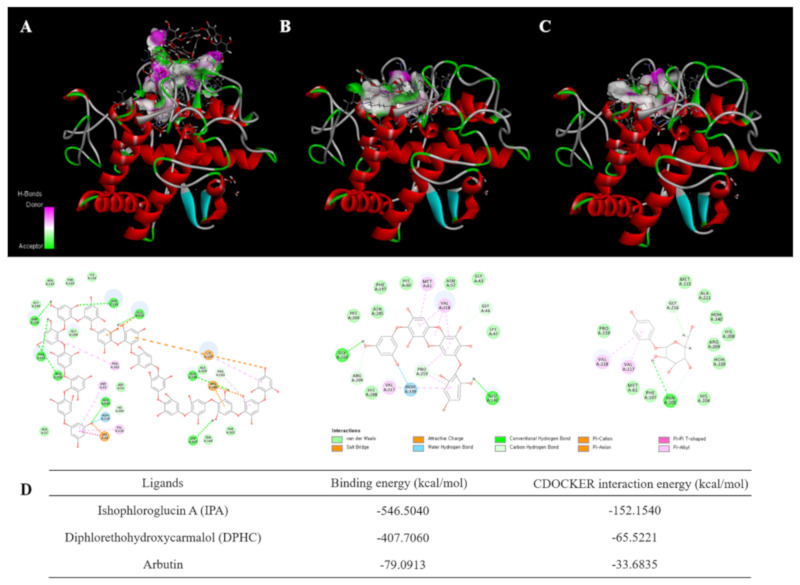
Computational prediction of the structure for tyrosinase and docking simulation with Ishophloroglucin A (IPA) and diphlorethohydroxycarmalol (DPHC). (**A**) Tyrosinase–IPA complex; (**B**) tyrosinase–DPHC complex; (**C**) tyrosinase–arbutin complex; (**D**) result of docking study of tyrosinase with IPA, DPHC and arbutin.

A docking method was carried out to simulate the binding of the commercial tyrosinase enzyme with IPA, DPHC, and arbutin [25]. The binding modes of IPA, DPHC, or arbutin with mushroom tyrosinase are presented in Figure 1A–C. As shown in the 3D diagram of the tyrosinase–IPA complex (Figure 1A), tyrosinase–DPHC (Figure 1B), and tyrosinase–arbutin (Figure 1C), although IPA, DPHC, and arbutin docked to the active site, the tyrosinase–IPA complex showed the largest binding surface area. In addition, as shown in a 2D diagram, the tyrosinase–IPA complex showed more interactions with the various amino acids of tyrosinase, compared to either tyrosinase–DPHC or tyrosinase–arbutin. Seven benzene rings (1st, 2nd, 3rd, 8th, 9th, 12th, and 16th) and their oxygen atoms have π–π interactions with Histidine60, Methionine61, Lysine157, Glutamate158, Proline160, Proline201, Arginine206, and Valine218. In particular, the 16th benzene ring was combined with Histidine60 and Histidine204, the main amino acids of the active site. In addition, many oxygen atoms in IPA interacted via hydrogen bonds with Glutamate158, Proline160, Aspartate167, Methionine184, Phenylalanine197, Asparagine199, Glutamine202, Asparagine205, and Arginine209. Furthermore, based on the docking analysis results, it was calculated that the total binding energy and CDOCKER interaction energy using the CDOCKER interaction energy program of DS 3.0 (Figure 1D) were as follows: interaction energy with tyrosinase: Ishophloroglucin A (IPA): −152.154 kcal/mol, diphlorethohydroxycarmalol (DPHC): −65.5221 kcal/mol and arbutin: −33.6835 kcal/mol and the binding energy with tyrosinase: Ishophloroglucin A (IPA): −546.504 kcal/mol, diphlorethohydroxycarmalol (DPHC): −407.706 kcal/mol and arbutin: −79.0913 kcal/mol.

### 2.2. Effects of Melanogenic Inhibitors on Melanin Synthesis in Zebrafish Larvae

**Figure 2:**
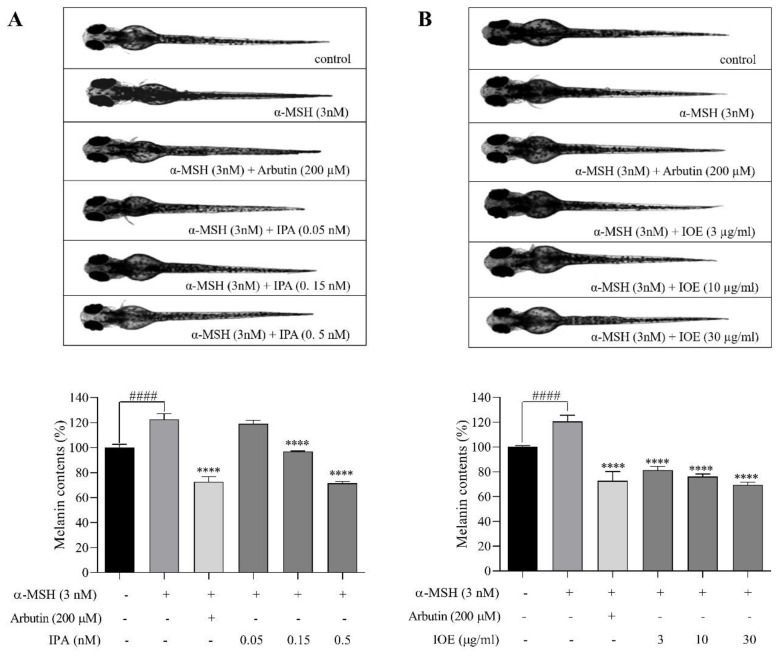
Morphological analysis on melanin synthesis in zebrafish larvae. Zebrafish embryos were treated with each melanogenic inhibitor from 7–9 h post-fertilization (hpf). The inhibitory effects of IPA and *Ishige okamurae* crude extract (IOE) on the pigmentation of zebrafish larvae were observed by microscopy, in IPA-treated zebrafish (**A**) and in IOE-treated zebrafish (**B**). Melanin content was determined. Arbutin is supplied as the positive control. The absorbance was measured at 490 nm. The concentrations of test samples were presented in graphs. Melanin content is expressed as percent values of control. Data are represented as means ± SD of independent experiments; ns, not significant; ^####^ *p* < 0.0001 compared to no sample treated group, **** *p* < 0.0001 compared to α-melanocyte-stimulating hormone (α-MSH)-treated group.

The purpose of this study was to determine whether IPA and IOE can inhibit melanogenesis in zebrafish larvae in vivo. In our previous study, IPA and IOE showed no toxicity in zebrafish larvae [26]. We treated zebrafish larvae with zebrafishes (0.05, 0.15, and 0.5 nM) and IOE (3, 10, and 30 μg/mL) to determine their melanin content. To estimate the inhibitory activities, we measured the total melanin content of the zebrafish extracts. The phenotypic effect on zebrafish melanin was observed by analyzing their body pigmentation under a microscope at room temperature. Surface melanin was significantly reduced by various concentrations of the target inhibitors (Figure 2). Treatment with 3 nM α-MSH significantly increased the melanin content of zebrafish compared with the untreated group. The highest concentrations of IPA (0.5 nM) and IOE (30 μg/mL) decreased the total melanin content by almost 28.56% and 30.36%, respectively, which presented similar effects as the arbutin (200 μM)-treated group (Figure 2A,B).

### 2.3. Effects of IPA and IOE on Melanin Synthesis in B16F10 Cells

**Figure 3:**
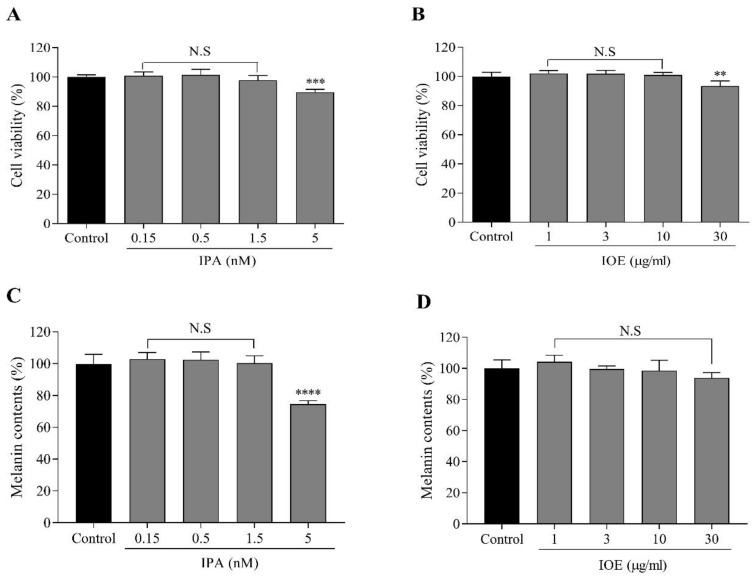
Effects of IPA and IOE on the cell viability and melanin content of B16F10 melanoma cells. Cytotoxicity of IPA (**A**), IOE (**B**) in B16F10 melanoma cells. Cells were incubated with different concentrations of IPA, IOE for 72 h and cell viability was determined by MTT (3-(4,5-dimethylthiazol-2-yl)-2,5-diphenyltetrazolium bromide) assay. Results are normalized to control. After 72-h incubation, absorbance was measured at 450 nm. Melanin content of group of IPA (**C**), group of IOE (**D**) in B16F10 cells. Melanin contents are expressed as percent values. The data are presented as means ± SD of independent experiments; ns, not significant; ** *p* < 0.01, *** *p* < 0.001, **** *p* < 0.0001 compared to no sample treated group.

The cytotoxic effects of IPA and IOE on B16F10 cells were measured via the MTT (3-(4,5-dimethylthiazol-2-yl)-2,5-diphenyltetrazolium bromide) assay. We initially examined the cytotoxic effects of IPA and IOE on B16F10 cells treated with different concentrations without α-MSH stimulation. IPA (0.15, 0.5, 1.5, and 5 nM) did not reveal any significant cytotoxicity on B16F10 cells, whereas IOE (1, 3, 10, and 30 μg/mL) showed no cytotoxicity (Figure 3A,B). The concentrations of IPA and IOE with no toxicity were used for further study.

We examined whether the effect of melanin inhibition from either IPA or IOE treatment in vivo was reversible without α-MSH stimulation in B16F10 cells. Melanin content was significantly decreased upon treatment with IPA (5 nM) or IOE (30 μg/mL) compared to the control group (Figure 3C,D). These results suggested that IPA and IOE inhibited melanin synthesis without α-MSH stimulation in B16F10 cells, thus contributing to its anti-melanogenesis effect.

### 2.4. Effects of IPA and IOE on Tyrosinase Activity and Melanin Synthesis in α-MSH-Stimulated B16F10 Cells

Hence, doses of IPA (0.5, 1.5, and 5 nM) and IOE (3, 10, and 30 μg/mL) were incubated with B16F10 melanoma cells to determine their bioactivity in cellular tyrosinase activity and α-MSH-mediated melanogenesis.

The concentrations of α-MSH and arbutin were determined based on the results of their cytotoxicity and melanin production in B16F10 cells (Figure S2A–D). By analyzing the data on survival rate, 1 nM of α-MSH and 100 μM of arbutin were used for further experiments.

**Figure 4:**
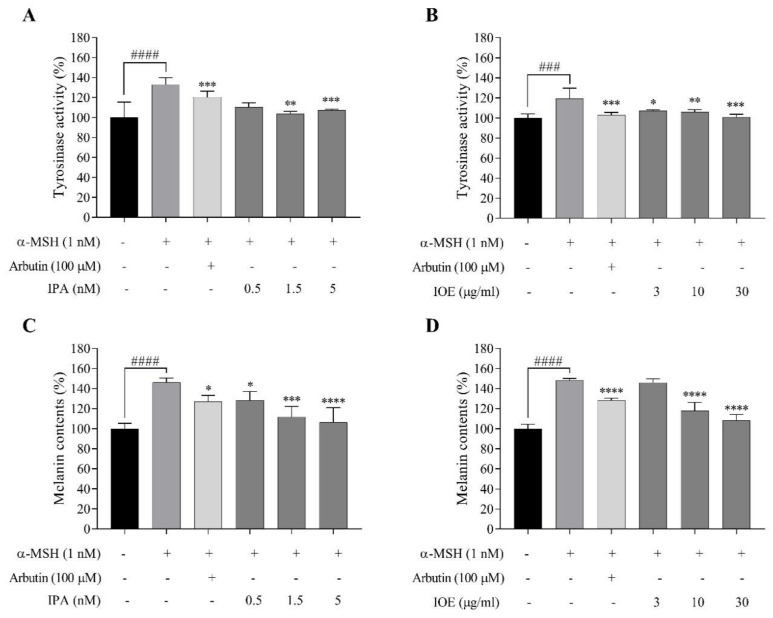
Effects of IPA (**A**) or IOE (**B**) on the tyrosinase activity in α-MSH-stimulated B16F10 melanoma cells; inhibitory effect of IPA (**C**) or IOE (**D**) on melanin production in B16F10 melanoma cells induced by α-MSH (1 nM). After 72-h incubation, absorbance was measured at 475 nm. Tyrosinase activity and melanin content expressed as percent values with the blank group as 100%. Data are compared with control group (the second bar) and represented as mean ± SD of independent experiments; ns, not significant; ^###^ *p* < 0.001, ^####^ *p* < 0.0001 compared to no sample treated group; * *p* < 0.05, ** *p* < 0.01, *** *p* < 0.001, **** *p* < 0.0001 compared to α-MSH-treated group.

As for the cellular tyrosinase activity, although the inhibitory effects of 1.5 nM of IPA were relatively lower than those depicted in the positive control, the concentration differed nearly 10 times, with the higher concentration of arbutin at 100 μM (Figure 4A). IOE reduced tyrosinase activity with a slight fluctuation between the effects of different concentrations compared to the α-MSH-treated group (Figure 4B). As for α-MSH-mediated melanogenesis, significant reductions in melanin content were observed in the 1.5 and 5 nM IPA-treated groups (Figure 4C). In addition, 30 μg/mL IOE inhibited pigmentation to a point that resembled the blank group (Figure 4D). These results suggest that IPA and IOE downregulated tyrosinase activity and that this inhibitory effect may lead to decreased cellular melanin synthesis in B16F10 cells.

### 2.5. Effects of IPA and IOE on the Molecular Mechanism in α-MSH-Stimulated B16F10 Cells

**Figure 5:**
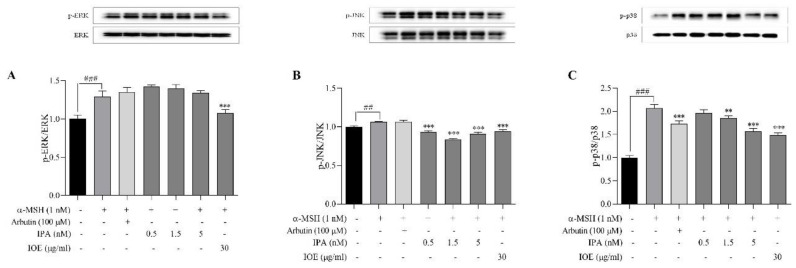
Effects of IPA and IOE on the expression of ERK (extracellular signal-regulated kinase), JNK (Jun N-terminal kinase), and p38 mitogen-activated protein kinase (MAPK)-mediated melanogenesis pathways in B16F10 cells induced by α-MSH. IPA and IOE were co-treated with α-MSH on B16F10 cells and cultured for 48 h. Representative photograph (**A**) and densitometric analysis (**B**,**C**) of Western blot analysis of ERK/p-ERK, JNK/p-JNK and p-p38/p38 proteins expression levels. The data were obtained from three independent experiments and values are presented as the means ± SD. ^##^ *p* < 0.01, ^###^ *p* < 0.001 compared to no sample treated group; ** *p* < 0.01, *** *p* < 0.001 compared with only α-MSH-treated group.

To elucidate the mechanism responsible for their melanogenesis inhibitory effect, we determined the influence of IPA and IOE on the expression levels of signaling molecules involved in melanin synthesis (Figure 5). ERK (extracellular signal-regulated kinase), JNK (Jun N-terminal kinase), and p38 belong to the mitogen-activated protein kinase (MAPK) intracellular signal transduction cascade [27,28]. As presented in Figure 5A,B, ERK phosphorylation was enhanced with IPA treatment, whereas the phosphorylation of JNK was slightly decreased after treatment with IPA (1.5 and 5 nM) or IOE (30 μg/mL). In addition, as presented in Figure 5C, p-p38 levels were highly increased in the α-MSH-stimulated groups, at around 80% compared to the control. Furthermore, after treatment with IPA, IOE, or arbutin, the phosphorylation of p38 was slightly suppressed. These findings suggest that the melanogenic inhibitory effects of IPA and IOE may be related to the JNK and p38 MAPK signaling pathways.

## 3. Discussion

The compound DPHC isolated from IOE already had reported tyrosinase inhibitory activity and protective effect against UV-B radiation-induced cell damage in vitro [8]; however, the anti-melanogenesis effect of the components derived from IOE in silico by interaction with tyrosinase, in vivo in phenotype studies in animal models, and their underlying molecular mechanisms, have not yet been examined. In the present study, we determined the anti-melanogenesis and tyrosinase inhibitory activities of IPA, a phlorotannin isolated from IO and IOE, in a vertebrate model of zebrafish in vivo and in B16F10 melanoma cells in vitro, after induction with α-MSH.

Treatment with α-MSH is known to induce melanin synthesis and tyrosinase activity [20]. In addition, tyrosinase has been demonstrated to be essential for melanogenesis [29]. Previous studies have shown that molecular docking can be used to evaluate tyrosinase inhibitory activity [30,31]. Thus, molecular docking calculations were performed to understand the binding model of IPA and DPHC, a known polyphenol isolated from IO, which has revealed a higher binding energy in anti-melanogenesis activity than arbutin, as a positive control. According to the results (Figure 1), IPA revealed the lowest docking scores, which indicated that the interaction of IPA with the target protein tyrosinase was stronger than the other two compounds, DPHC and arbutin. However, there is a limitation in correlating the inhibition of mushroom tyrosinase activity with that of cellular tyrosinase or melanin production in cultured melanocytes [32]. Thus, the inhibitory effects of IPA on tyrosinase activity and melanogenesis were examined in a zebrafish in vivo model and murine B16F10 melanoma cells.

Melanin pigments accumulate on the surface of zebrafish, allowing microscopic observation of the pigmentation process without complicated experimental procedures, making them a suitable model for screening melanogenesis inhibitors [33,34]. We evaluated the melanin inhibitory effects of IPA and IOE in a zebrafish larva model stimulated by α-MSH through melanin content determination. All the tested samples exerted profound inhibitory effects on zebrafish pigmentation with no significant toxicity (Figure S2). The inhibitory effects of pigmentation were observed via morphological analysis of zebrafish larvae related to the different treatments (Figure 2). In addition, the use of early stage larvae rather than the adult stage provides another advantage of testing the percutaneous effects of medicinal or cosmetic compounds [33,35]. In this case, we chose α-MSH as an inducer both in zebrafish in vivo and in B16F10 melanoma cells in vitro. According to both results in zebrafish embryos and in B16F10 melanoma cells, the melanin content increased by α-MSH stimulation.

Melanin content correlates directly with the activity and protein levels of tyrosinase [36]. Therefore, we determined the inhibitory effects of IPA and IOE on the tyrosinase activity induced by α-MSH on B16F10 cells. We found that the IPA-treated group had a decreased tyrosinase activity and melanin content stimulated by α-MSH, with a reduction of approximately 35% tyrosinase activity and 40% melanin content (Figure 4A,C). IOE inhibited the activity of tyrosinase in a dose-dependent manner and significantly decreased melanogenesis in B16F10 cells (Figure 4B,D). Compared with arbutin, IPA and IOE have significant inhibitory effects on melanin production and tyrosinase activity, which was in accordance with the results of previous molecular docking studies.

To investigate the mechanism of the inhibitory effects on α-MSH-induced melanin synthesis in cells, Western blotting was performed. The expression levels of melanin-related proteins, including ERK, JNK, and p38, following treatment with IPA or IOE, were evaluated. Activated phosphorylation of ERK can promote the degradation of MITF via the ubiquitin–proteasome-dependent pathway [28]. This suggests that potential melanogenesis inhibitors may suppress melanin synthesis by promoting the proteasomal degradation of MITF, which was related to the activation of the ERK signaling pathways [37]. The ERK, JNK, and p38 MAPKs belong to the MAPK family [38,39,40]. In addition, activation of the p38 MAPK pathway induced MITF expression [41]. In this study, Western blot analysis revealed that IPA promotes p-JNK and p-p38 (Figure 5B,C). In contrast, the levels of p-ERK did not change with the treatment of IPA and IOE (Figure 5A). These results imply that the inhibitory effects of IPA and IOE on tyrosinase activity and melanogenesis may be related to the JNK and p38 signaling pathways.

## 4. Materials and Methods

### 4.1. Chemicals and Reagents

Dimethylsulfoxide (DMSO), 3-(4,5-dimethylthiazol-2-yl)-2,5-diphenyltetrazolium bromide (MTT), L-DOPA, alpha-melanocyte stimulating hormone (α-MSH) and phosphate buffered saline (PBS) were purchased from Sigma–Aldrich Chemical Co. (St. Louis, MO, USA). Dulbecco’s modified Eagle’s medium (DMEM) and fetal bovine serum (FBS) were obtained from Invitrogen–Gibco (Grand Island, NY, USA). The extracellular signal-regulated kinase (ERK1/2), phosphorylated ERK1/2 (p-ERK1/2), c-Jun N-terminal kinase (JNK), phosphorylated JNK (p-JNK), p38, phosphorylated p38 (p-p38), cAMP response element-binding protein (CREB), phosphorylated CREB (p-CREB), microphthalamia-associated transcription factor (MITF), tyrosinase-related protein-1 (Trp-2), tyrosinase-related protein-1 (Trp-1), tyrosinase (TYR), anti-mouse and anti-rabbit IgG antibodies were purchased from Cell Signaling Technology (Beverly, MA, USA). All other reagents, including α-MSH were purchased from Sigma–Aldrich Chemical Co.

### 4.2. Molecular Docking of Tyrosinase

For the docking study, the crystal structure of tyrosinase (PDB: 3NM8) was obtained from the Protein Data Bank (PDB, http://www.pdb.org). The docking studies were performed using CDOCKER in Accelrys Discovery Studio 3.0 (Accelrys, Inc., San Diego, CA, USA). When a whole nucleotide sequence is covered by the receptor grid, ligands are presumed to select the best docking position [42]. The docking procedure was mentioned in the previous study. Briefly, three steps were followed: (1) conversion of the 2D structure into a 3D structure; (2) calculation of charges; and (3) addition of hydrogen atoms using the flexible docking program [31,43].

### 4.3. Preparation of IOE and Isolation of IPA

IO was harvested in June 2018 along the east coast of Jeju Island, Korea. The alga was washed twice with tap water to remove the salt, epiphytes, and sand attached to the surface. Next it was carefully rinsed with fresh water, and was maintained in a medical refrigerator at −20 °C. Thereafter, the frozen alga was lyophilized and homogenized with a grinder prior to extraction. The IOE extracted in 50% ethanol (*v*/*v*, in water) under stirring for 24 h at room temperature, then it was filtered. The filtrated extract was concentrated under decompression and freeze-dried to powder (IOE). A 50% ethanol extract of IO was conducted by Shinwoo Co. Ltd. (Lot No. SW9E29SA, Gyeonggi-do, Korea). IPA was isolated from IOE as previously described [9]. Briefly, the IOE was fractionated using centrifugal partition chromatography. All fractions were collected and the IPA was eventually purified by a semi-preparative HPLC column (YMC-Pack ODS-A, 10 mm, 250 mm, 5 m). IPA was determined as a polyphenol and its chemical structure (Figure S1, Supplementary Materials) was identified via LC/MS analysis with a mass *m/z* of 992.1315, thus indicating a molecular formula of C_96_H_66_O_48_ (1986.26 of calculated molecular weight, Δ0.6, [M − 2H] ^2−^).

### 4.4. Origin and Maintenance of Parental Zebrafish

Adult zebrafish were obtained from a commercial dealer (Seoul Aquarium, Seoul, Korea) and 10 fish were preserved in a 3-L acrylic tank at 28.5 °C, with a 14:10 h light:dark cycle. Zebrafish were fed twice a day, for 6 days⁄week, with supplementary Tetramin flake food (SEWHAPET Food Co., Seoul, Korea). Embryos were collected within 30 min by natural spawning and induced in the morning by turning on the light. The zebrafish experiment received approval from the Animal Care and Use Committee of Jeju National University (Approval No. 2017-0001).

### 4.5. Measurement of Melanin Content in Zebrafish Larvae

IPA and IOE and stimulator α-MSH concentrations were used in order to examine the effects of concentration on the embryo development. Fifteen zebrafish embryos (3–4 hpf) were seeded in each well, comprising 1.9 mL embryo medium, in a 12-well seeding plate. Test samples were dissolved in 1% DMSO with 1× PBS and mixed well. Every morning for the first 5 dpf, viable embryos were enumerated in order to obtain a survival measure. To determine the melanin content, embryos at 7–9 hpf were seeded into a 6-well plate with 30 embryos in each well into a 2.7-mL embryo medium. After 3 days, the larvae were rinsed twice with 1× PBS in order to remove any residual reagents or particles and similar amounts of larvae were placed into the e-tubes. Before measuring the melanin content, several larvae of each group were captured with a microscope and remaining were centrifuged.

After centrifugation, the pellet was dissolved in 1 mL of 1 N NaOH at 90 °C for 60 min. The mixture was then vigorously vortexed to solubilize the melanin pigment. Absorbance of the supernatant was measured at 490 nm. The result was compared with the control which was considered to represent one hundred. The melanin content was calibrated by protein amount, and the observations were repeated in triplicate.

### 4.6. Cytoxicity of IPA and IOE in B16F10 Cells

B16F10 mouse melanoma cells were obtained from ATCC (American Type Culture Collection, Manassas, VA, USA). B16F10 cells were cultured in DMEM supplemented with 100 U/mL of penicillin, 100 μg/mL of streptomycin and 10% FBS. The cells were then incubated in an atmosphere of 5% CO_2_ at 37 °C and were then sub-cultured every 3–5 days. The cytotoxicity of IPA and IOE against B16F10 cells were investigated via colorimetric MTT assay. Briefly, the cells were seeded in 24-well plates at a concentration of 2 × 10^4^ cells/mL. About 16 h after seeding, the cells were incubated with IPA and IOE at different concentrations for 72 h, and their viability was determined.

### 4.7. Determination of Cellular Melanin Content

Cellular melanin content was measured using a previously described method [33]. The cells (2 × 10^4^ cells/mL) were incubated with various concentrations of IPA and IOE for 72 h; therefore, they were washed in ice-cold PBS. Briefly, the cells were incubated at 80 °C for 1 h in 1 mL of 1 N NaOH/10% DMSO and were then vortexed to solubilize the melanin: the absorbance was measured at 450 nm. The optical density of the inhibition in the control was considered to be 100%. The data are presented in the form of mean percentages and the results were repeated in triplicate.

### 4.8. Tyrosinase Inhibition Activity and Melanin Content Induced by α-MSH

Cellular tyrosinase activity was measured according to the previously reported method with slight modifications [33]. Briefly, the cells were cultured at 2 × 10^4^ cells/mL in 24-well plates.

About 16 h after cells seeding, the cells (2 × 10^4^ cells/mL) were stimulated with α-MSH (1 nM) and were then incubated with IPA and IOE for 72 h. The cells were washed with PBS and lysed in PBS containing 1% Triton X-100 by freezing and thawing. The lysates were clarified by centrifugation at 13,000 rpm for 10 min. After protein quantification and normalization, 90 μL of cell lysate (each sample contained the same amount of protein) was incubated in duplicate with 10 μL of 10 mM L-DOPA at 37 °C for 1 h. After incubation, dopachrome was monitored by measuring the absorbance at 475 nm using the ELISA reader. The value of each measurement is expressed as the percentage change from the control.

### 4.9. Western Blot Analysis

B16F10 cells were treated with indicated concentrations of IPA or IOE. The cells were collected and suspended in a lysis buffer (150 mM NaCl, 50 mM Tris-HCl (pH 7.5), 5 mM EDTA, and 1% Triton X-100) containing protease inhibitors (170 μg/mL leupeptin and 100 μg/ mL PMSF). After incubating at 4 °C for 20 min, the cell lysates were centrifuged at 12,000 rpm for 10 min. Each cell supernatant was collected for protein concentration measurement with a bicinchoninic acid protein assay kit (Thermo Scientific, Waltham, MA, USA). Proteins (20 μg) were separated by 10% SDS (Sodium Dodecyl Sulfate)-polyacrylamide gel electrophoresis (SDS-PAGE), and were then transferred onto nitrocellulose membranes (Bio-Rad, Hercules, CA, USA). These membranes were then blocked by Tris-buffered saline-Tween 20 solutions (TBS-T) containing 5% non-fat dry milk, incubated with primary antibodies at 4 °C for 24 h, washed with TBST, and incubated with secondary antibodies at room temperature for 2 h. Protein bands were visualized using an ECL detection kit and a luminescent image analyzer (LAS-3000, Fujifilm, Tokyo, Japan).

### 4.10. Statistical Analysis

All data are presented as the mean ± standard deviation (SD) of three determinations. The mean values were statistically compared by analysis of one-way (ANOVA) multiple comparisons, followed by Dunnett’s multiple comparisons test using Graphpad prism 7 software. A value of *p* < 0.05 level was considered as statistically different.

## 5. Conclusions

In conclusion, IPA, isolated from IOE, inhibits tyrosinase activity and melanogenesis induced by α-MSH in vivo and in vitro. These results indicated that IPA derived from IOE showed potential for critical interaction in the active site of tyrosinase, which reversibly reduces pigmentation in zebrafish larvae in vivo, and mechanistically works via modulating JNK and p38 MAPKs in α-MSH-induced B16F10 cells. Although further study is needed for IPA isolated from IOE with an appropriate array of tests using human models for its use as a therapeutic or cosmetic agent, this study suggests that IPA is a potential candidate for the treatment of hyperpigmentation and other related diseases.

## Supplementary Materials

The following are available online at https://www.mdpi.com/1660-3397/18/9/470/s1, Figure S1. Structure of Ishophloroglucin A (IPA, A) and Diphlorethohydroxycarmalol (DPHC, B) isolated from Ishige okamurae. Figure S2: Effects of α-MSH and arbutin on the cell viability and melanin content of B16F10 melanoma cells. Cytotoxicity of α-MSH (A) and arbutin (B) in B16F10 melanoma cells. Cells were incubated with different concentrations of α-MSH 0.1, 0.3, 1, 3, and 10 nM) and arbutin (10, 30, 100, and 300 μM) for 72 h and cell viability was determined by MTT assay. Results are normalized to control. Melanin contents of group of α-MSH (C) and group of arbutin (D) in B16F10 cells. After 72 h incubation, absorbance was measured at 450 nm. Melanin contents are expressed as percent values. The data are shown as means ±SD of independent experiments; ns, not significant; ^*^*p* < 0.05, ^**^*p* < 0.01, and ^***^*p* < 0.001 compared to no sample treated group.
