# Supplementary material for: Ishophloroglucin A Isolated from Ishige okamurae Suppresses Melanogenesis Induced by α-MSH: In Vitro and In Vivo
Source: Mar Drugs. 2020 Sep 17;18(9):470. doi: 10.3390/md18090470 (PMC7551695; doi:10.3390/md18090470)
Supplement: Supplementary file 1 [file marinedrugs-18-00470-s001.pdf]

marine drugs

Supplementary Material

Ishophloroglucin A Isolated from *Ishige okamurae* Suppresses Melanogenesis Induced by α-MSH: In Vitro and In Vivo

Xining Li [1], Hye-Won Yang [1], Yunfei Jiang [1], Jae-Young Oh [1], You-Jin Jeon [1,2,*] and Bomi Ryu [1,*]

1 Department of Marine Life Sciences, Jeju National University, Jeju 63243, Korea; lixn887@nenu.edu.cn (X.L.); koty221@naver.com (H.-W.Y.); jiangyunfei0310@gmail.com (Y.J.); ojy0724@naver.com (J.-Y.O.)
2 Marine Science Institute, Jeju National University, Jeju 63333, Korea
* Correspondence: youjinj@jejunu.ac.kr (Y.-J.J.); bmryu@jejunu.ac.kr (B.R.); Tel.: +82-64-754-3475 (Y.-J.J. & B.R.)

Figure S1. Structure of Ishophloroglucin A (IPA, A) and Diphlorethohydroxycarmalol (DPHC, B) isolated from *Ishige okamurae*

Figure S2. Effects of α-MSH and arbutin on the cell viability and melanin content of B16F10 melanoma cells. Cytotoxicity of α-MSH (A) and arbutin (B) in B16F10 melanoma cells. Cells were incubated with different concentrations of α-MSH 0.1, 0.3, 1, 3, and 10 nM) and arbutin (10, 30, 100, and 300 μM) for 72 h and cell viability was determined by MTT assay. Results are normalized to control. Melanin contents of group of α-MSH (C) and group of arbutin (D) in B16F10 cells. After 72 h incubation, absorbance was measured at 450 nm. Melanin contents are expressed as percent values. The data are shown as means ±SD of independent experiments; ns, not significant; $^{*}p < 0.05$, $^{**}p < 0.01$, and $^{***}p < 0.001$ compared to no sample treated group.

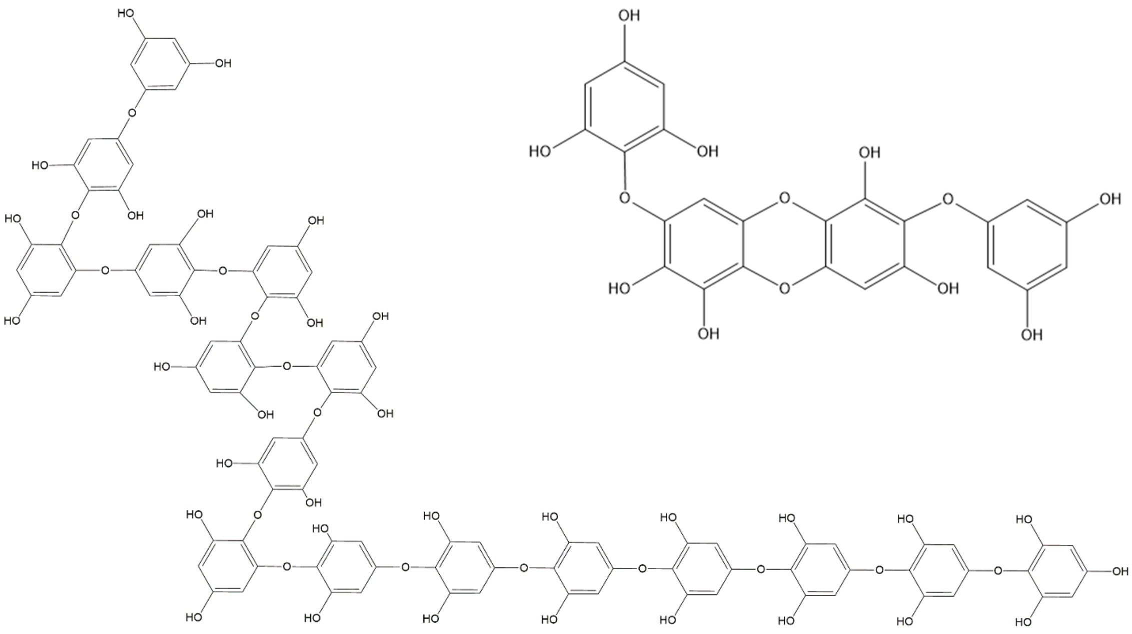

A	B

Figure S1.

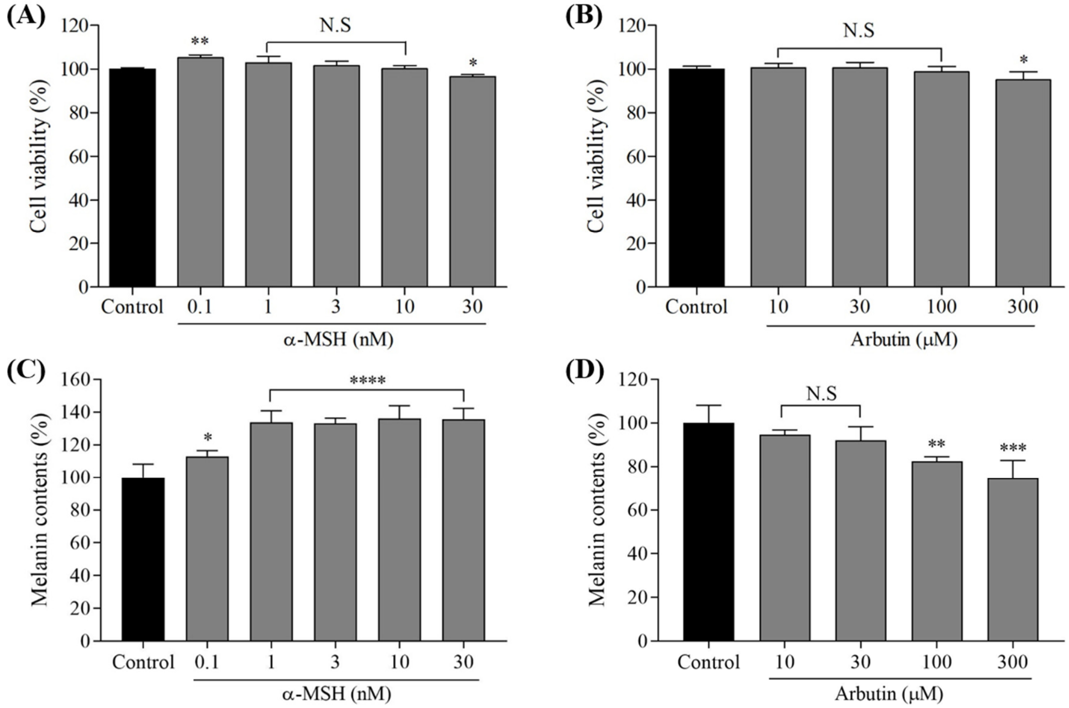

Figure S2.

© 2020 by the authors. Licensee MDPI, Basel, Switzerland. This article is an open access article distributed under the terms and conditions of the Creative Commons Attribution (CC BY) license (http://creativecommons.org/licenses/by/4.0/).
